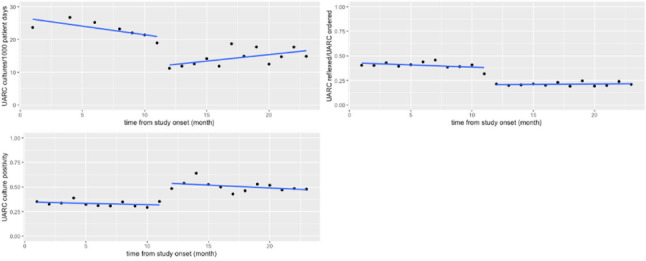# Effecting the culture: Impact of changing urinalysis with reflex culture criteria on culture rates and outcomes

**DOI:** 10.1017/ash.2022.79

**Published:** 2022-05-16

**Authors:** Jessica Penney, Angie Rodday, Paola Sebastiani, David Snydman, Shira Doron

## Abstract

**Background:** Urinalysis and urine culture are frequently ordered diagnostic tests among hospitalized patients, often for nonspecific symptoms. Diagnostic testing stewardship with urinalysis with reflex culture (UARC) is a practice shown to reduce institutional culture rates by selecting patients who are more likely to have a true infection. Optimal reflex criteria are not well established, and downstream effects, such as antibiotic use, have not been well studied. **Methods**: We compared outcomes in the preimplementation period (December 2018 – October 2019) and postintervention period (November 2019–October 2020) at an academic medical center. The intervention was changing the UARC reflex criteria. The primary outcomes were urine-culture rate per 1,000 patient days, urine-culture positivity, antibiotic prescription for suspected urinary tract infection (UTI) and catheter-associated urinary tract infection (CAUTI) rate per 1,000 Foley catheter days. Analysis was performed using interrupted time-series negative binomial regression or Poisson regression where appropriate. **Results:** We detected a significant decrease in the rate of cultures performed (32.5 cultures per 1,000 patient days before the intervention vs 8.6 cultures per 1,000 patient days after the intervention; *P* = 0.10). Fig. 1 summarizes these results graphically. In an adverse events analysis, of 646 patients in the postintervention period, 130 patients were reviewed for the outcome of sepsis secondary to a urinary tract infection, with only 1 patient meeting criteria for this diagnosis. **Conclusions:** Changing the UARC reflex criteria resulted in the expected decrease in rate of cultures performed with increase in culture positivity, and the stricter criteria appeared to more effectively identify true UTIs. Minimal adverse events were associated with the UARC criteria change, demonstrating that these criteria are also safe. We detected a significant change in antibiotic prescriptions, but much of the decrease occurred during the preintervention period, which likely reflected educational and stewardship interventions performed at that time. Although the intervention affected culture performance, which does decrease institutional costs, continued provider education is needed to influence clinical outcomes.

**Funding:** None

**Disclosures:** None

**Figure:**